# Cranial nerve IV palsy after mild head trauma: a rare image

**DOI:** 10.11604/pamj.2021.39.156.11232

**Published:** 2021-06-29

**Authors:** Hassen Ben Ghezala

**Affiliations:** 1 Service Universitaire des Urgences et de Réanimation Médicale, Hôpital Régional de Zaghouan, Faculté de Médecine de Tunis, Tunis, Tunisie

**Keywords:** Trochlear nerve, palsy, brain injury, trauma

## Image in medicine

Mild traumatic brain injury is characterized by a Glasgow coma scale GCS between 13 and 15. It is a frequent pathology in our country because of the increasing number of circulation accidents. Trauma-induced superior oblique palsy usually results from contusion or avulsion of the trochlear nerve. Severe craniocerebral trauma is often associated with the former mechanism, whereas more minor closed-head injuries can decompensate a congenital phoria. We report a patient who developed an isolated trochlear nerve palsy following minor head trauma. Our 53 years-old patient without significant past medical history was victim of a traffic accident. He presented with a complaint of diplopia following head trauma the week prior. He lost consciousness for less than 1 minute. He noticed diplopia immediately upon awakening, but had no other neurologic symptoms. He presented to our emergency department for evaluation. Computed brain tomography (CT) performed did not show any orbital fractures. Ocular exam revealed mild to moderate infraduction deficit and supraduction excess in adduction of the left eye. Left head tilt test was negative. We finally concluded that our patient had a trochlear nerve palsy following the brain trauma. Further Magnetic Resonance Imaging (MRI) revealed an unsuspected tentorial vascular malformation that was compressing the trochlear nerve in its subarachnoid course. In the absence of other features that support decompensation of a congenital trochlear nerve palsy, compressive lesions should be sought in case of fourth cranial nerve palsy following head trauma.

**Figure 1:**
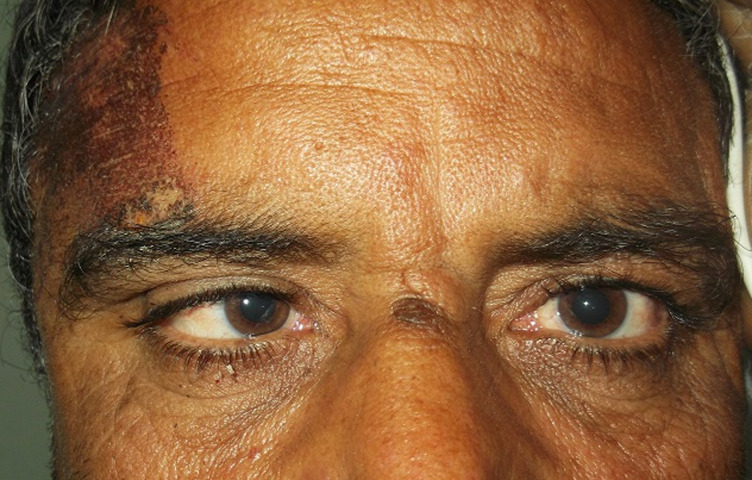
paralysis of adduction of the left eye

